# Comorbidity patterns in cardiovascular diseases: the role of life-stage and socioeconomic status

**DOI:** 10.3389/fcvm.2024.1215458

**Published:** 2024-02-13

**Authors:** Héctor A. Cruz-Ávila, Fernando Ramírez-Alatriste, Mireya Martínez-García, Enrique Hernández-Lemus

**Affiliations:** ^1^ Graduate Program in Complexity Sciences, Autonomous University of México City, México City, Mexico; ^2^ Immunology Department, National Institute of Cardiology ‘Ignacio Chávez’, México City, Mexico; ^3^ Computational Genomics Division, National Institute of Genomic Medicine, México City, Mexico; ^4^ Center for Complexity Sciences, Universidad Nacional Autónoma de México, México City, Mexico

**Keywords:** comorbidities, cardiovascular diseases, human disease network, diseasome, socioeconomic status, health care

## Abstract

Cardiovascular diseases stand as a prominent global cause of mortality, their intricate origins often entwined with comorbidities and multimorbid conditions. Acknowledging the pivotal roles of age, sex, and social determinants of health in shaping the onset and progression of these diseases, our study delves into the nuanced interplay between life-stage, socioeconomic status, and comorbidity patterns within cardiovascular diseases. Leveraging data from a cross-sectional survey encompassing Mexican adults, we unearth a robust association between these variables and the prevalence of comorbidities linked to cardiovascular conditions. To foster a comprehensive understanding of multimorbidity patterns across diverse life-stages, we scrutinize an extensive dataset comprising 47,377 cases diagnosed with cardiovascular ailments at Mexico’s national reference hospital. Extracting sociodemographic details, primary diagnoses prompting hospitalization, and additional conditions identified through ICD-10 codes, we unveil subtle yet significant associations and discuss pertinent specific cases. Our results underscore a noteworthy trend: younger patients of lower socioeconomic status exhibit a heightened likelihood of cardiovascular comorbidities compared to their older counterparts with a higher socioeconomic status. By empowering clinicians to discern non-evident comorbidities, our study aims to refine therapeutic designs. These findings offer profound insights into the intricate interplay among life-stage, socioeconomic status, and comorbidity patterns within cardiovascular diseases. Armed with data-supported approaches that account for these factors, clinical practices stand to be enhanced, and public health policies informed, ultimately advancing the prevention and management of cardiovascular disease in Mexico.

## 1 Introduction

Cardiovascular diseases in diferent life-stages constitute big challenges for health systems worldwide. Most of these conditions may be related to ageing, sex and socioeconomic status (SES) and are also strongly associated with the particular complex patient’s needs, social determinants and quality of healthcare (2, 1). Patients may complicate in their evolution by multiple cooccuring medical conditions or comorbidities making more challenging to care for them (2). To account for this, the term *comorbidity* was coined to represent the occurrence of other medical conditions in addition to an index condition of interest (3).

Such comorbidity relationships occur whenever two or more diseases are present in the same individual more often than by chance alone (4, 5). Multimorbidity is associated with the risk of premature death, loss of functional capacity, depression, complex drug regimes, psychological distress, declined quality of life, increase in hospitalizations and decreased productivity. This is also linked to economic burden for health-care systems and society (7, 6).

There is evidence linking comorbidity with social determinants of health (SDHs) such as cultural issues, social support, housing, demographic environment and SES. Together, these factors, make a source of complexity that creates potential vulnerability for someone facing it, many of them in disavantage for being in socioeconomically deprived areas. The association between SES and prevalence of multimorbidity has been recently established (8, 11, 10, 9, 12). Also, these complex phenomena have profound implications for the delivery of high quality care of chronic health conditions and remark the necesity of complex interventions to tackle multimorbidity (2, 13–15).

Likewise, the non-random co-occurrence of certain diseases differs in different life-stages: the prevalence of multimorbidity increases with age, 60% of the events are reported among 65–74 year olds higher than the prevalence of each individual disease (16, 17). Also, the sex is increasingly perceived as a key determinant of multimorbidity in cardiovascular disease (CVD). Although it has been seen as a predominantly men disease, due to men’s higher absolute risk compared with women, the relative risk in women of CVD morbidity and mortality is higher increased by some medical factors (diabetes, hypertension, hypercholesterolemia, obesity, chronic kidney, rheumatoid arthritis and other inflammatory joint diseases) (18, 19). Moreover, serious multiple chronic conditions are more common in older women (rather than 65) and may limit treatment alternatives (10, 20, 21).

Multimorbidity presents many challenges, which may at times seem overwhelming. In such a scenario, evidence-based treatment guidelines, designed for single diseases, may lead to serious therapeutic conflicts (1). To circumvent such limitations, a personalized approach to medicine may benefit from the inclusion of ideas from a somewhat recent field of research, generally known as *systems biology* or *network medicine* –when applied to humans–. This approach offers the potential to decipher and understand the relationships between comorbidities at a much deeper level by considering coordinated instances (systems) rather than single conditions. A theoretical framework of diseasome has indicated that most human diseases are interdependent. These concept lead to anoher called the *human disease network* (HDN), a graph in which two diseases are connected if they have in common some biological, genetic, metabolic o even socioeconomic element (1).

The present study is aimed to analyze the patterns of cardiovascular-associated multimorbidity stratified by life-stages, sex and socioeconomic status. Studying such patterns at a large scale will be useful, both to discover trends helpful for public health care planning, as well as to serve as additional clues to understand the complex interaction between genetic/molecular, clinic and social/environmental conditionants of cardiovascular diseases in the different age/sex/SES.

Here, we will expand upon the outcomes derived from various comorbidity networks under consideration. These networks were constructed based on previously outlined criteria, taking into account structural characteristics arising from relationships between pairs of diseases. The focus will be on *mutual information* (MI) shared by diseases (depending on the frequency of their joint presence, as we will see later), acting as an indicator of co-occurrence between two conditions. This approach offers an additional perspective for discerning comorbidity patterns across diverse networks.

To pinpoint comorbidities potentially linked to sex and/or SES within each network, we will employ the *Page Rank Score* (PRS), a network indicator of overall influence. This scoring system enables the numerical identification of diseases with greater relevance within each network (1). By factoring in MI between pairs of diseases, the PRS enhances precision, providing richer insights into comorbidity and multimorbidity phenomena in individuals.

The main reserach question that we will aboard in this work is then *How does the interplay between life-stage and socioeconomic status influence the comorbidity patterns in cardiovascular diseases among the Mexican metropolitan population, and what are the subtle associations and differences in comorbidity prevalence across age groups and socioeconomic strata?*

## 2 Materials and methods

### 2.1 Data acquisition (electronic health records)

The National Institute of Cardiology ’Ignacio Chávez’ (NICICH), one of Mexico’s National Institutes of Health, is the reference hospital for specialized cardiovascular care in Mexico. The NICICH it is also a third level hospital receiving in-patients with related ailments such as metabolic, inflammatory and systemic diseases, whose treatment may involve immunology, rheumatology, nephrology, and similar specialities in addition to cardiology-related treatments (1).

**Figure 1:**
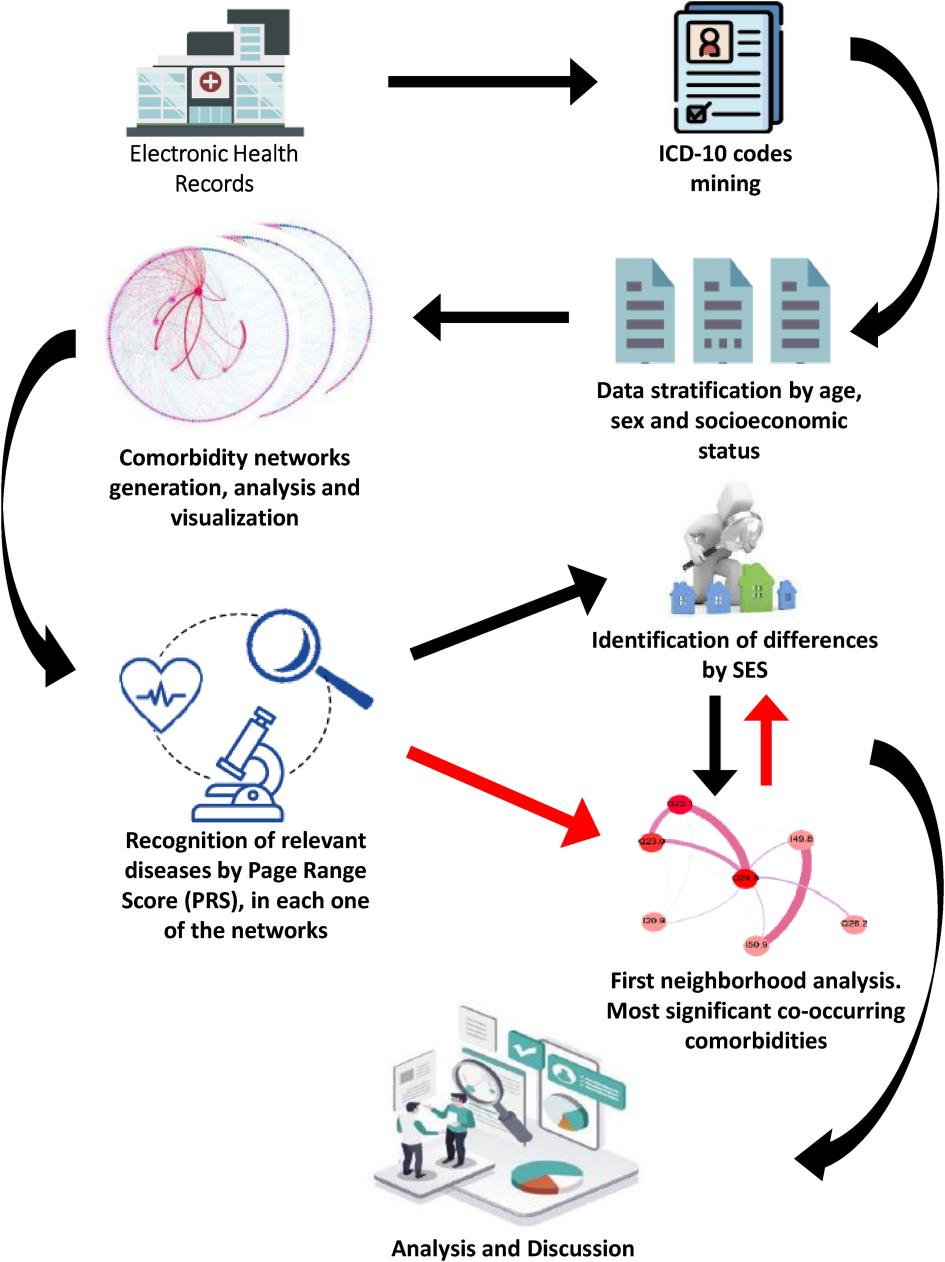
Flow chart of study. The figure represents the general workflow of this study from data collection (EHR mining), data pre-processing and processing, analytics, results and discussion.

In this work we used the NICICH Electronic Health Record (EHR) database entries as recorded between January 1, 2011 and June 31, 2019. The EHR-database contains information on socioeconomic factors as well as the main clinical diagnosis that led to hospitalization, it also reports other diseases, disorders, conditions or health problems that the individuals may present. The SES as recorded in the institutional file is a well-defined construct that involves the weighting of variables related to education, employment status, family monetary income, access to public services (water, electricity, drainage) and housing conditions (rural or urban). The EHR management procedures of the institution are set to provide up to five main comorbidities. International Classification of Diseases, tenth revision (ICD-10) was used to identify and clasificate them. The full set of hospital discharged patients, with all types of diagnostics, age, sex and SES were considered in the time period under study, with the exception of those with incomplete information or erroneous coding. The study population included 47, 377 discharged cases. The cardiovascular comorbidities assessed included any disease registered in each case (see Figure 1).

### 2.2 Data processing (ICD-10 coding)

Once EHR data has been pre-processed to tabular format, disease and comorbidity relationships could be investigated. Mining, processing and cross-transforming ICD-10 data were performed using the icd (v. 4.0.9) R library (22) (https://www.rdocumentation.org/packages/icd/versions/4.0.9). While ICD codes are increasingly becoming useful tools in the clinical and basic research arenas, their use is not free of caveats and limitations (for a brief dicsussion of some of these in the context of current norms, please refer to the relevant paragrpahs in the discussion section).

### 2.3 Statistical analysis

A database of 47, 377 electronic health records (EHRs) was used as this study corpus. Analysis was stratified by age and sex group. Descriptive statistics were used to summarize overall information. The chronic conditions with the highest prevalence, stratified by SES, and the number of chronic conditions associated with each disease were computed.

#### 2.3.1 Cohort stratification

For the purpose of statistical and network analysis, patients were stratified based on age and sex. The age groups were defined as follows: the 0–20 years old age bracket has 9,782 individuals (20.65%) of which 4,921 are women and 4,861 men; for the 21–40 years old range there were 6,939 individuals (14.65%) split into 3,593 women and 3,346 men; the 41–60 year old range included 13,690 persons (28.90%) with 5,095 women and 8,595 men; 14,537 (30.68%) individuals conformed the 61–80 years old group with 5,695 women and 8,842 men; lastly the 81 years and older group had 2,429 (5.13%) registered patients 1,187 women and 1,242 men. These strata were used to build the different comorbidity networks that will be presented and discussed later.

#### 2.3.2 Cardiovascular comorbidity network (CVCnetworks)

Electronic health records data was processed using in-house developed code (in the R programming language) for the design and analysis of comorbidity networks as previously reported (1). Programming code for this study is available in the following public access repository: https://github.com/CSB-IG/Comorbidity_Networks. Once the mining of the medical cases was carried out, a set of *undirected networks* (one network for each age/sex/SES bracket combination, see Subsection 2.4), was built based on the significant co-ocurrent diseases coded according to ICD-10 codes.

Briefly, the origin and destination nodes in these networks are diseases as identified with their respective ICD-10 codes. Subsequently, a link was drawn between these nodes, as long as at least these diseases co-occurr in the same person within this group more often than by chance alone (*hypergeometric test*, with a False Discovery Rate (FDR) multiple testing correction *FDR* < 0.05). The strength of the comorbidity association was determined by using the MI calculated for each pair of diseases in the CVCnetworks by using a custom made script (available at https://github.com/CSB-IG/ICD_Comorbidity/blob/main/Disc_Mut_Info.py) based on the mutual_info_score function of the sklearn.metrics Python package.

### 2.4 Network statistics and visualization

In network theory, one of the parameters used to evaluate the connections in the graph is the *degree centrality* (DC), the total number of links on a node or the sum of the frequencies of the interactions. The degree distribution of a disease is the number of ICD-10 codes associated with that disease. Aside from the node degree, a relevant centrality measure is the PRS (23) that captures the relative influence of a given node in the context of network communication. The Network Analyzer plugin (24) in the Cytoscape open source network analysis suite was used to explore and visualize the network (25) and also the CytoNCA package was used to calculate further network centrality measures (26).

*Betweenness centrality* (BC) measure is used to assess the relevance of a given condition in the context of a node’s influence in global network information flow. Weighted network analytics, PRS calculations and visualization were performed by using Gephi (27). In brief, MI will be used to assess the *strength* of comorbidity relations (i.e., a higher MI value represents a stronger comorbidity association between two diseased conditions). PRS on the other hand will be used to assess the relevance of a given disease in the context of the comorbidity network given its vicinity (i.e., a higher PRS value represents a higher potential to become a multimorbid condition). In the context of this vicinity, we will often refer to the set of diseases *directly* connected to a given disease as their comorbidity nearest neighbors (CNNs).

In this study, a double-circle layout visualization was implemented, where nodes were arranged according to their PRS in a counter-clockwise direction, and the top 10 highest ranking diseases were placed in the center of the graph. Nodes were colored on a gradient scale from red (higher closeness centrality) to blue (lower closeness centrality). Additionally, the node size was determined based on their Betweenness Centrality measure, where larger nodes indicated a higher value of Betweenness Centrality.

## 3 Results

### 3.1 Cardiovascular comorbidity networks general results

Comorbidity networks were built for the specific age/sex/SES as previosuly described and a general topological analysis was conducted prior to a detailed analysis of each network. Table 1 present the main topological features of these networks. By examining the connectivity and structural patterns, significant relationships can be identified, which will be discussed later (A set of tables containing the full connectivity informations for all the networks can be found in the Supplementary Materials).

**Table 1:** Topological characteristics of comorbidity networks by age, sex and socioeconomic status (low or high).

Age, sex and SES bracket	Nodes	Links	Average number of neighbors	Clustering coefficient	Network centralization	Network density
Men						
Low ≤ 20	408	2,593	12.832	0.756	0.591	0.032
High ≤ 20	195	1,034	10.74	0.764	0.721	0.056
Low 21–40	617	3,727	12.233	0.693	0.468	0.02
High 21–40	204	739	7.307	0.726	0.446	0.036
Low 41–60	619	5,316	17.232	0.729	0.374	0.028
High 41–60	309	1,708	11.055	0.768	0.474	0.036
Low 61–80	589	5,097	17.337	0.8	0.47	0.03
High 61–80	334	2,434	14.575	0.755	0.442	0.044
Low ≥81	193	1,256	13.016	0.725	0.421	0.068
High ≥81	175	944	10.789	0.737	0.42	0.062
Women						
Low ≤20	443	2,715	12.313	0.746	0.579	0.028
High ≤20	183	947	10.35	0.738	0.626	0.057
Low 21–40	675	4,038	12.084	0.717	0.449	0.018
High 21–40	222	763	6.995	0.727	0.411	0.032
Low 41–60	693	5,320	15.601	0.717	0.4	0.023
High 41–60	289	1,399	7.749	0.741	0.526	0.034
Low 61–80	570	4,996	17.643	0.754	0.434	0.031
High 61–80	341	2,069	12.135	0.76	0.446	0.036
Low ≥81	255	1,634	12.909	0.752	0.36	0.051
High ≥81	158	901	11.405	0.72	0.507	0.073

The analysis of the various networks shown in Table 1 revealed that, overall, individuals with low SES exhibited a higher diversity of diseases, reflected in the larger number of nodes, often double or more compared to high SES networks. This phenomenon is attributed to health inequalities arising from constraints faced by this population, making them more susceptible to developing diseases not prevalent in high SES individuals or manifesting and being treated differently due to varying access to necessary resources, ranging from adequate nutrition to healthcare access.

The clustering coefficient showed notable uniformity across all networks, with the network corresponding to men aged 61–80 with low SES exhibiting the highest clustering level. This suggests a higher likelihood of individuals in this network easily manifesting any disease within the network from an initial disease. While this observation doesn’t sharply differentiate from other networks, it raises concerns about disease interactions and, consequently, treatment concerning pharmacological interactions.

The higher prevalence of disease diversity in low SES may be associated with the greater density observed in high SES networks. This increased density implies more interconnections among all diseases in high SES networks. However, this doesn’t necessarily indicate a higher propensity for comorbidity in high SES individuals, as evidenced by a considerably higher number of connections in low SES, particularly among men over 80.

Furthermore, finding a higher network centralization in graphs for the age range of 0–20 years, with even greater centralization in high SES, may result from comorbidities influenced by factors related to birth. Additionally, there is a lower disease diversity in high SES, while the higher number of diseases in low SES diversifies the conditions centralizing comorbidity relationships.

The average number of comorbidities, measured through the average neighbors in various networks, tends to be higher in older ages compared to young individuals. This trend is more pronounced in men than women. Notably, the decrease in the average number of comorbidities in the population over 80 contradicts existing literature.

**Figure 2:**
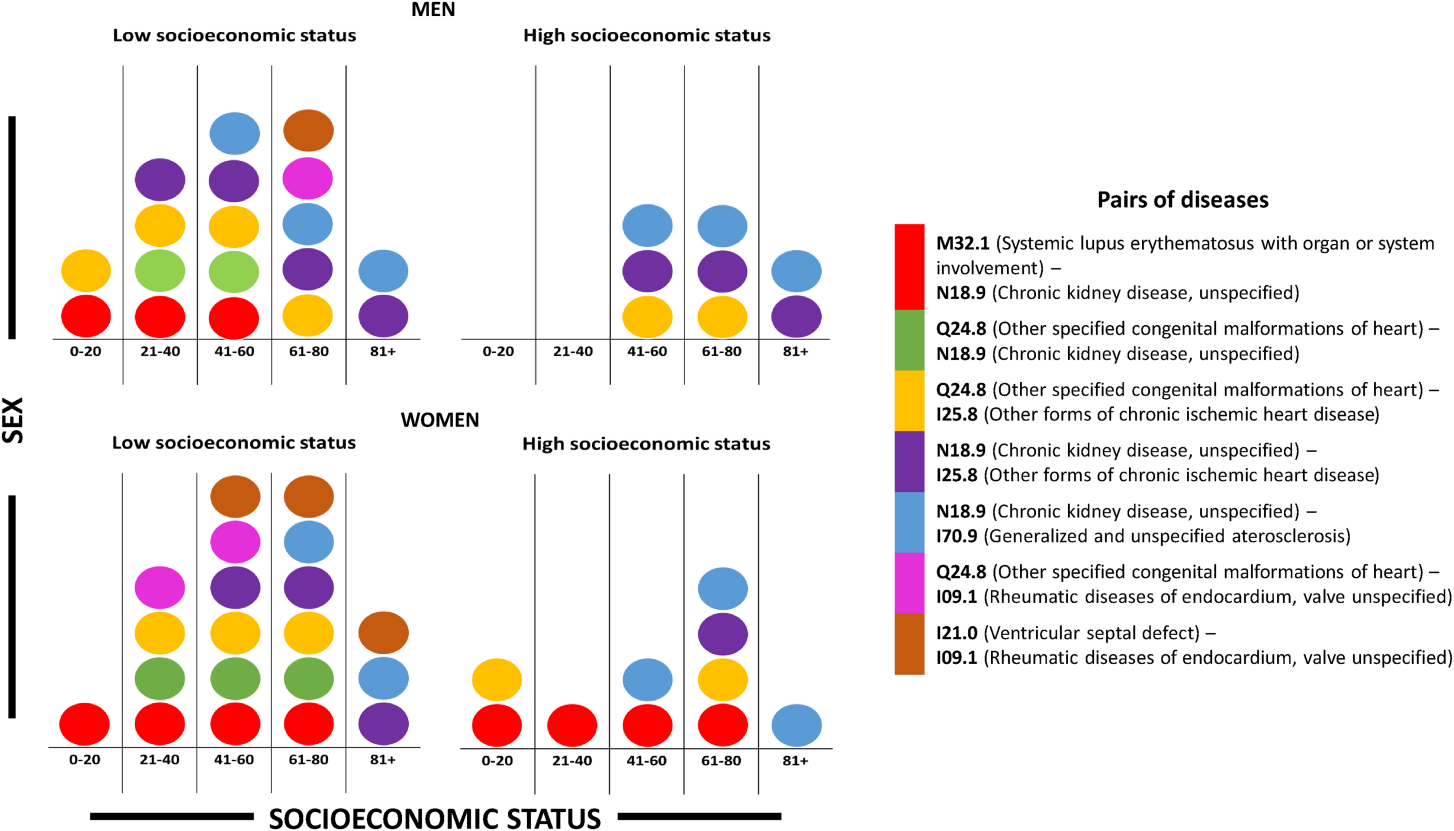
Presence of disease pairs in different stages of life by sex and socioeconomic status.

A closer analysis of the different CVC networks reveal that there are some *pairs of diseases* that are prevalent as the most relevant comorbidities in more than one group of age/sex/SES. Some of the most relevant of these pairs of diseases are shown in Figure 2. Understanding disease pairs that transcend *demographic* (e.g., age/sex/SES) boundaries may help us to provide a holistic view of health challenges and opportunities for intervention. It may contribute to more effective public health strategies and policies that consider the interconnected nature of diseases across diverse populations. Let us examine some of the implications.

### 3.2 Cardiovascular comorbidity networks based on socioeconomic status in men and women aged 20 years or younger

**Figure 3:**
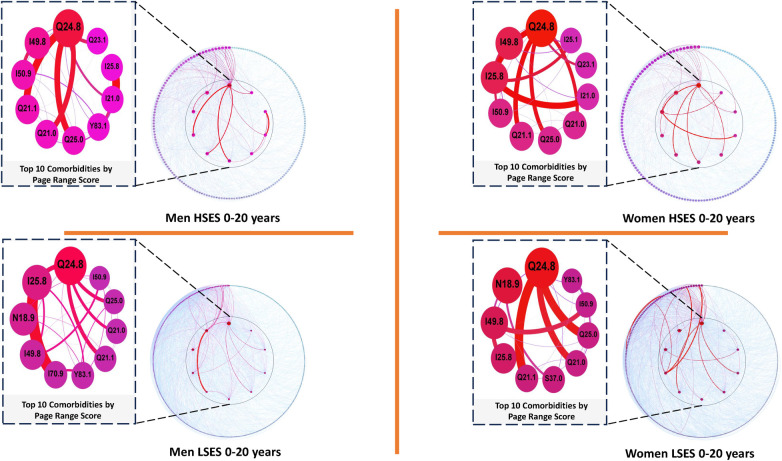
Comorbidity networks for patients aged 0–20 years old, for both sexes in the low (LSES) and high (HSES) socioeconomic status. Nodes are ordered according with their Page Rank Score (PRS). High PRS nodes appear in the center. Size and color intensity (red implies higher values, blue lower values) of the nodes is also given by the PRS as a measure of relative importance in the network. Size and color of the edges represent the mutual information weight among disease pairs.

Upon visual inspection of the distinct networks depicted in Figure 3, discernible structural differences in their connectivity patterns come to light. A more nuanced examination of the network statistics could provide additional insights into shared features and commonalities. For example, Table 2 highlights the top 5 diseases with the highest comorbidity burden, as indicated by their respective PRS within the specified network.

**Table 2:** Top 5 diseases with a higher comorbidity burden in networks for men and women patients of low and high SES aged 20 years old or less as well as their PRS value.

M-LSES		W-LSES		M-HSES		W-HSES	
Node	Pagerank	Node	Pagerank	Node	Pagerank	Node	Pagerank
Q24.8	0.122383	Q24.8	0.123032	Q24.8	0.203587	Q24.8	0.186019
I25.8	0.066727	N18.9	0.060586	I49.8	0.056417	I49.8	0.065737
N18.9	0.051396	I49.8	0.042086	I50.9	0.040504	I25.8	0.063193
I49.8	0.036886	I25.8	0.032545	Q21.1	0.038370	I50.9	0.034251
I70.9	0.026809	Q21.1	0.023382	Q21.0	0.033428	Q21.1	0.033435

Several commonalities emerge among these highly comorbid diseases. Regardless of sex or SES, the notable presence of *Other specified congenital malformations of the heart* (Q24.8) and *Generalized and unspecified atherosclerosis* (I70.9) is observed. Additionally, *Other forms of chronic ischemic heart disease* (I25.8) appears in three out of four networks, except for men with high SES, where it ranks 9th according to its PRS. A similar scenario unfolds for *Atrial septal defect*, which moves to the 7th rank in men of low SES. Notably, most highly prevalent and comorbid diseases in this age group exhibit a strong genetic risk component, likely explaining their consistently high rankings across all four networks, irrespective of sex or SES.

Equally noteworthy, albeit for divergent reasons, are instances such as *Unspecified cardiac insufficiency* (I50.9), which holds a high rank solely among individuals of both sexes with high SES, and *Chronic kidney disease, unspecified* (N18.9), appearing exclusively in the top 5 for men and women of low SES. The association of unspecified chronic disease with low SES in children and young adults (up to 20 years old) suggests a probable link to environmental factors. Consequently, we opted to investigate its network neighborhood. Intriguingly, robust comorbidity relationships with M32.1 *Systemic lupus erythematosus with organ or system involvement* were identified in networks corresponding to different age/sex/SES categories.

It is pertinent to note that the documented presence of what has been termed *lupus nephritis* in children is well-established (29–31, 28). Notably, lupus nephritis can be specifically reported using the ICD-10 code M32.14 *Glomerular disease in systemic lupus erythematosus*, rather than the more general code M32.1. Nevertheless, the occurrence of juvenile systemic lupus erythematosus (JSLE) has been reported as a more active disease in children and young adults, characterized by faster progression and worse outcomes, including progressive chronic kidney disease, compared to its adult-onset counterpart, leading to poorer long-term survival. Studies indicate that lupus nephritis may affect up to 50%–75% of all children with JSLE. Consequently, analyzing the comorbidity landscapes associated with either concurrent N18.9 and M32.1 (or M32.14) may offer valuable insights for determining optimal diagnostic and therapeutic strategies to enhance patient outcomes.

### 3.3 Cardiovascular comorbidity networks based on socioeconomic status in men and women aged 21–40 years

**Figure 4:**
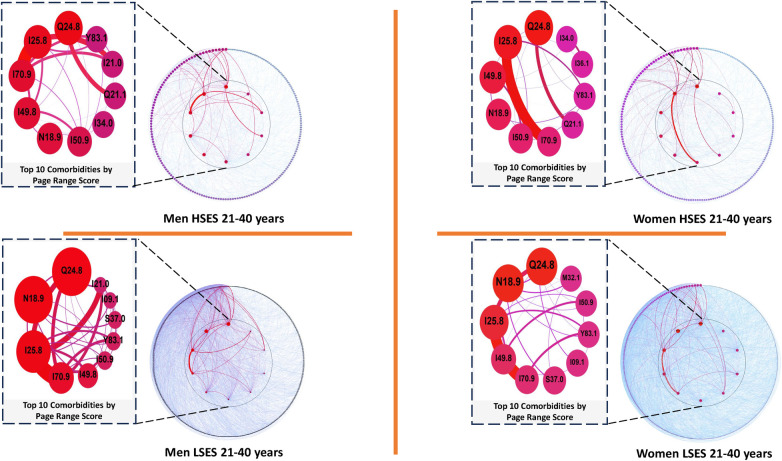
Comorbidity networks for patients aged 21–40 years old, for both sexes in the low (LSES) and high (HSES) socioeconomic status. Visualization parameters are as in Figure 3.

Examination of comorbidity networks for individuals aged 21–40 years, encompassing both sexes and SES, reveals similar trends in highly comorbid conditions as observed in children and young adults (aged 0–21 years). Noteworthy diseases, including *Other specified cardiac arrhythmias* (I49.8), *Other forms of chronic ischemic heart disease* (I25.8), *Chronic kidney disease, unspecified* (N18.9), and *Other specified congenital malformations of the heart* (Q24.8), consistently rank among the top 5 conditions with high PRS in their respective networks, irrespective of sex or SES (see Table 3 and Figure 4).

**Table 3:** Top 5 diseases with a higher comorbidity burden in networks for men and women patients of low and high SES aged 21 through 40 years old as well as their PRS value.

M-LSES		W-LSES		M-HSES		W-HSES	
Node	Pagerank	Node	Pagerank	Node	Pagerank	Node	Pagerank
Q24.8	0.075559	Q24.8	0.076926	Q24.8	0.107942	Q24.8	0.102099
IN18.9	0.073513	N18.9	0.075713	I25.8	0.081587	I25.8	0.079363
I25.8	0.064252	I25.8	0.047414	I70.9	0.058656	I49.8	0.053809
I70.9	0.043823	I49.8	0.028615	I49.8	0.055704	N18.9	0.042437
I49.8	0.031521	I70.9	0.024069	N18.9	0.049351	I50.9	0.033641

It is evident that, up to this age bracket, the most highly morbid conditions are largely shared across different SES. Notably, *Other and unspecified atherosclerosis* (I70.9), which does not appear in the top 5 for women with high SES in Table 3, is nonetheless ranked 6th in that particular subgroup.

### 3.4 Cardiovascular comorbidity networks based on socioeconomic status in men and women aged 41–60 years

**Figure 5:**
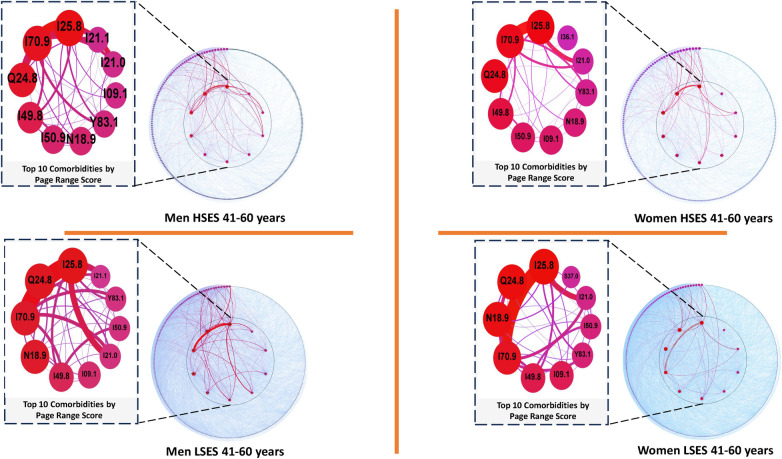
Comorbidity networks for patients aged 41–60 years old, for both sexes in the low (LSES) and high (HSES) socioeconomic status. Visualization parameters are as in Figure 3.

In examining the networks for the current age range (depicted in Figure 5), notable diseases consistently rank among the top five in each graph, as outlined in Table 4. *Other forms of chronic ischemic heart disease (I25.8)*, *Other specified cardiac arrhythmias (I49.8)*, *Other specified congenital malformations of the heart (Q24.8)*, and *Unspecified atherosclerosis (I70.9)* persist in both men and women across all SES. Conversely, *Unspecified heart failure (I50.9)* exclusively appears in the high SES group, while *Unspecified chronic kidney disease (N18.9)* emerges solely among the five most relevant diseases in the low SES group. Consequently, a more in-depth analysis of these latter two diseases was undertaken.

**Table 4:** Top 5 diseases with a higher comorbidity burden in networks for men and women patients of low and high SES aged 41 through 60 years old as well as their PRS value.

M-LSES		W-LSES		M-HSES		W-HSES	
Node	Pagerank	Node	Pagerank	Node	Pagerank	Node	Pagerank
I25.8	0.082013	I25.8	0.073004	I25.8	0.109405	I25.8	0.086848
Q24.8	0.067377	Q24.8	0.063748	I70.9	0.081470	I70.9	0.069631
I70.9	0.057113	N18.9	0.058209	Q24.8	0.069968	Q24.8	0.067638
N18.9	0.054875	I70.9	0.047838	I49.8	0.046008	I49.8	0.046347
I49.8	0.031014	I49.8	0.029453	I50.9	0.031122	I50.9	0.035720

Regarding *Unspecified heart failure (I50.9)* in the low SES group, it maintains a substantial position, ranking eighth among both men and women based on their PRS. In contrast, *Unspecified chronic kidney disease (N18.9)* continues to feature prominently in the high SES group, ranking sixth among men and seventh among women according to their PRS.

As no discernible differences were observed in SES based on the PRS, a first-neighbors analysis was conducted on the diseases listed in Table 4. This analysis considered the MI between pairs of diseases and examined the relationships forming between the diseases mentioned in the table. Notably, the relationship between *Unspecified atherosclerosis (I70.9)* and *Unspecified chronic kidney disease (N18.9)*, sharing an MI of 0.018057, exhibited a distinction with respect to SES in women networks.

### 3.5 Cardiovascular comorbidity networks based on socioeconomic status in men and women aged 61–80 years

In this population, Table 5 highlights consistent representation of the same diseases among the top positions in all graphs, including *Other forms of chronic ischemic heart disease (I25.8)*, *Unspecified atherosclerosis (I70.9)*, *Other specified congenital malformations of the heart (Q24.8)*, and *Other specified cardiac arrhythmias (I49.8)*. Notably, *Unspecified chronic kidney disease (N18.9)* ranks sixth for high-SES men. Similarly, *Unspecified rheumatic diseases of endocardial valve (I09.1)* appears in sixth place for low-SES men and women across both strata.

**Table 5:** Top 5 diseases with a higher comorbidity burden in networks for men and women patients of low and high SES aged 61 through 80 years old as well as their PRS value.

M-LSES		W-LSES		M-HSES		W-HSES	
Node	Pagerank	Node	Pagerank	Node	Pagerank	Node	Pagerank
I25.8	0.095870	I25.8	0.084714	I25.8	0.109745	I25.8	0.104393
I70.9	0.066529	Q24.8	0.059171	I70.9	0.081787	I70.9	0.071043
Q24.8	0.058011	I70.9	0.059062	Q24.8	0.056171	I49.8	0.044746
N18.9	0.042366	N18.9	0.050231	I49.8	0.037394	Q24.8	0.043568
I49.8	0.032505	I49.8	0.030941	I09.1	0.031442	N18.9	0.038826

**Figure 6:**
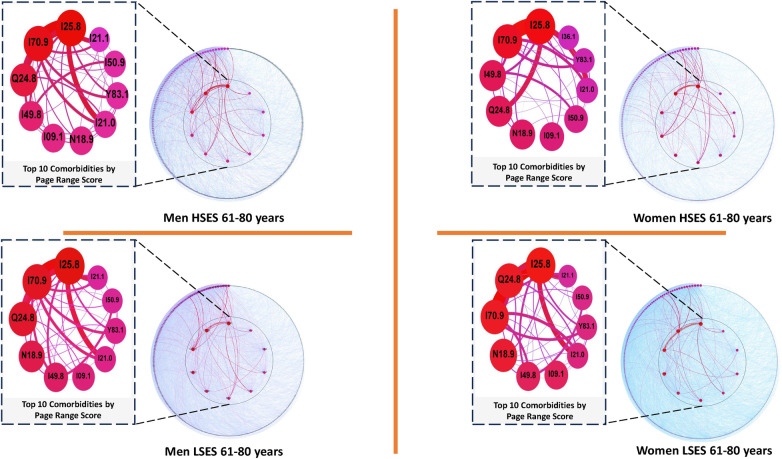
Comorbidity networks for patients aged 61–80 years old, for both sexes in the low (LSES) and high (HSES) socioeconomic status. Visualization parameters are as in Figure 3.

In the analysis of nearest neighbors, it was found that *Other specified congenital malformations of the heart (Q24.8)*, consistently positioned in the networks from early stages of life, is linked to *Unspecified rheumatic diseases of endocardial valve (I09.1)* exclusively in low-SES men, sharing an MI of 0.015235 and occupying the fifty-second position among the relationships in this population. This phenomenon appears solely in this age range and in low-SES men (see Figure 6). For women, the relationship between *Other specified congenital malformations of the heart (Q24.8)* and *Unspecified rheumatic diseases of endocardial valve (I09.1)*, absent in the present age range, is evident between 21 and 60 years old, exclusively in the low-SES group.

### 3.6 Cardiovascular comorbidity networks based on socioeconomic status in men and women aged 80 and older

**Figure 7:**
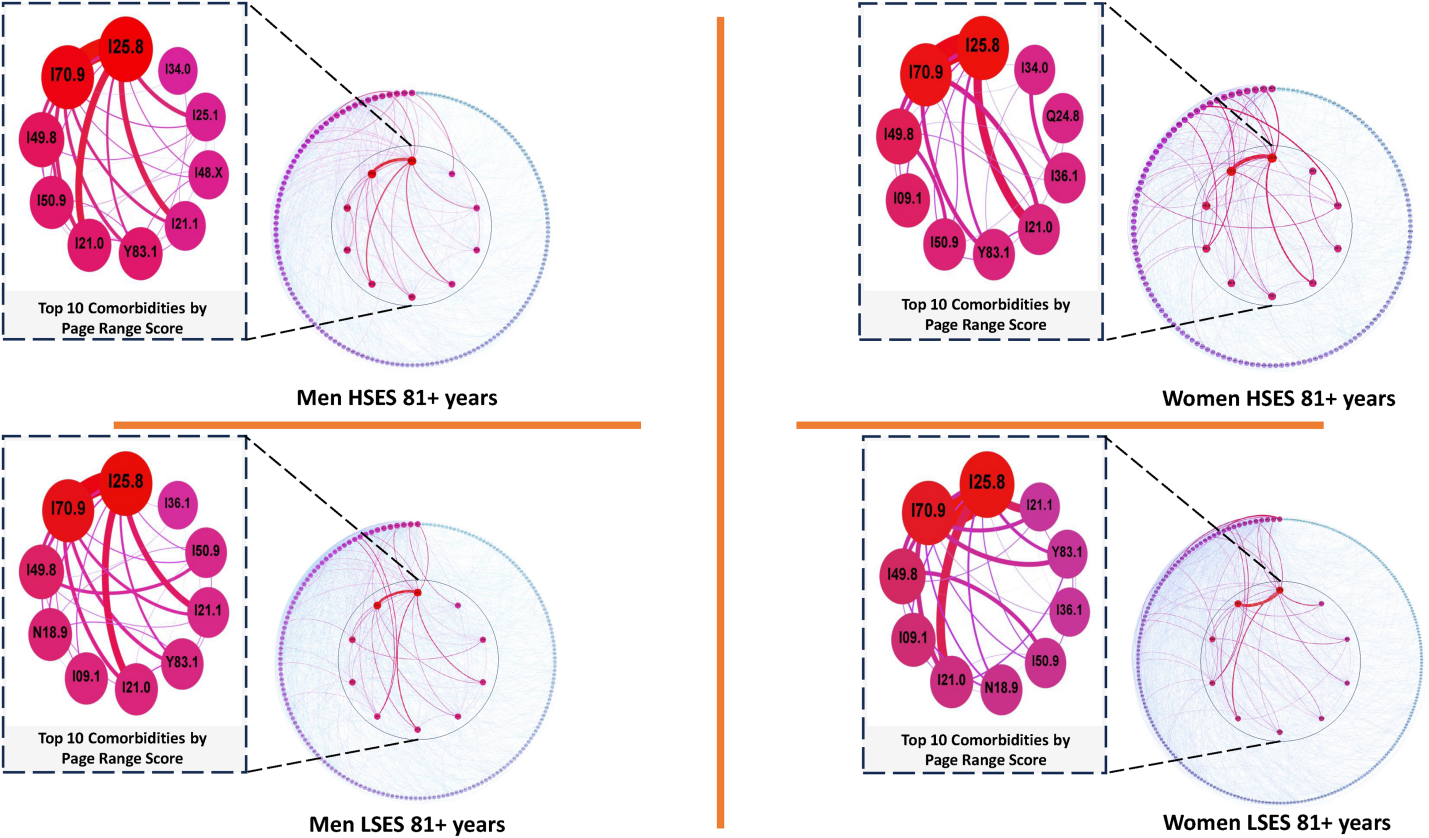
Comorbidity networks for patients aged 81 years and older, for both sexes in the low (LSES) and high (HSES) socioeconomic status. Visualization parameters are as in Figure 3.

In the population aged 80 and older, Table 6 reveals a consistent top three diseases across both sexes and SES (see Figure 7): *Other forms of chronic ischemic heart disease (I25.8)*, *Unspecified atherosclerosis (I70.9)*, and *Other specified cardiac arrhythmias (I49.8)*. These conditions maintain their prominence throughout the lifespan of the study population, alongside *Unspecified heart failure (I50.9)* and *Unspecified chronic kidney disease (N18.9)*.

**Table 6:** Top 5 diseases with a higher comorbidity burden in networks for men and women patients of low and high SES aged 80 years and older as well as their PRS value.

M-LSES		W-LSES		M-HSES		W-HSES	
Node	Pagerank	Node	Pagerank	Node	Pagerank	Node	Pagerank
I25.8	0.135882	I25.8	0.115066	I25.8	0.148629	I25.8	0.125256
I70.9	0.098356	I70.9	0.080434	I70.9	0.108438	I70.9	0.099107
I49.8	0.040327	I49.8	0.038061	I49.8	0.043204	I49.8	0.044240
N18.9	0.033998	I09.1	0.032181	I50.9	0.035299	I09.1	0.037596
I09.1	0.031539	I21.0	0.028179	I21.0	0.035240	I50.9	0.030817

Regarding the latter two conditions, it is noteworthy that *Unspecified heart failure (I50.9)*, absent among the top five diseases in the low SES group, ranks ninth for men and seventh for women in this stratum. On the other hand, *Unspecified chronic kidney disease (N18.9)*, exclusive to men in the low SES group, is positioned nineteenth for men in the high SES group. For women, it appears sixth in the low SES group and twelfth in the high SES group according to their PRS.

As for the remaining two diseases, they exhibit a relationship that is exclusive to women in the low SES group within the present age range. In this co-occurrence, the pair ranks ninety-sixth out of 1,634 disease pairs, with a MI of 0.004605. It is noteworthy that, at the individual level, *Acute transmural myocardial infarction of anterior wall (I21.0)*, which, according to the IPR analysis, is absent in women of high SES and men of low SES, takes the seventh place in the former case and the sixth place in the latter. Conversely, *Unspecified rheumatic diseases of endocardium valve (I09.1)* holds the thirteenth place of relevance for men of high SES.

The relationship between *Acute transmural myocardial infarction of anterior wall (I21.0)* and *Unspecified rheumatic diseases of endocardium valve (I09.1)*, observed in previous age ranges, is limited to the low SES group. Specifically, it appears only between the ages of 61 and 80 for men and from 41 up to the present age range for women.

## 4 Discussion

In this section, we will delve deeper into the outcomes derived from the various comorbidity networks, which were constructed based on the previously described criteria considering the structural characteristics arising from relationships between pairs of diseases. The analysis incorporates mutual information as an indicator of co-occurrence between two diseases, offering a supplementary perspective for discerning comorbidity patterns within these networks.

Shared pairs of diseases across several ge/sex/SAS strata (recall Figure 2) may provide information in the following dimensions:

• **Cross-sectional patterns:** Identifying disease pairs across different demographic groups can highlight patterns that persist across various segments of the population. This suggests that certain diseases may interact or co-occur consistently, irrespective of age, sex, or SES.
• **Public health implications:** Disease pairs present in multiple demographic groups may have broader public health implications. Understanding these commonalities can guide healthcare policies, resource allocation, and preventive measures that are applicable to a diverse range of individuals.
• **Targeted interventions:** Recognizing consistent disease pairs allows for the development of targeted interventions that address shared risk factors or mechanisms. It enables healthcare providers to implement more effective and tailored strategies for prevention, early detection, and management.
• **Complex interactions:** The presence of shared disease pairs across demographic groups may indicate complex interactions between diseases that go beyond individual factors. Investigating these interactions can provide insights into the underlying mechanisms and pathways leading to comorbidities.
• **Longitudinal health monitoring:** Tracking disease pairs across different life stages and demographics contributes to a more comprehensive understanding of health trajectories. It facilitates the identification of critical points where interventions can be most impactful in preventing the development of severe comorbidities.
• **Reducing health disparities:** Recognizing common disease pairs across diverse populations helps in addressing health disparities. It allows for a more equitable distribution of healthcare resources and interventions, aiming to reduce the disproportionate burden of certain diseases on specific demographic groups.
• **Research and policy focus:** Disease pairs that persist across age, sex, and SES groups may warrant further research attention and policy focus. They can serve as indicators for the need to explore underlying determinants, social factors, and genetic influences contributing to the observed patterns.

The identification of comorbidities potentially associated with sex and/or SES in each network involved the utilization of the Page Rank Score. This numerical measure allows us to pinpoint diseases with greater relevance within each network (1). The PRS enhances precision by considering the MI between pairs of diseases, thereby providing more detailed insights into comorbidity and multimorbidity phenomena in individuals.

### 4.1 Comorbidity networks: general observations

In examining the disparities across the analyzed networks, a noteworthy observation emerges: individuals with low SES generally exhibit a greater diversity of diseases, often double or more, compared to their high-SES counterparts. This pattern suggests that individuals with low SES face health inequalities, making them more susceptible to a broader spectrum of diseases. These diseases may either not occur in high-SES individuals or manifest differently, influenced by varying access to essential resources for their care—ranging from nutrition to healthcare services (34, 33, 32).

Moreover, the heightened diversity of diseases in the low-SES group may be linked to the greater density observed in high-SES networks. This increased density results in more connections between all diseases in high-SES networks. However, despite the higher number of connections, individuals with low SES exhibit a significantly higher number of comorbidity relationships, as evident from Table 1.

The finding of greater network centralization, particularly in the age range of 0–20 years—accentuated in high SES during these ages—may be attributed to specific comorbidities mediated by factors related to birth. This narrower diversity of diseases in high SES during these ages suggests distinct comorbidity patterns. Additionally, the greater number of diseases in low SES contributes to diversifying the conditions centralizing comorbidity relationships. Factors unique to low SES, such as overcrowding, nutrition, and structural conditions during infancy, expose individuals to different health challenges, potentially leading to varied patterns of comorbidity and multimorbidity in the short or long term (5, 36, 35).

Similarly, our analysis revealed that the average number of comorbidities, measured by the average number of neighbors in the different networks, is higher in older age groups compared to younger individuals (refer to Table 1). However, this trend is more pronounced in men than in women, as indicated by our results. Notably, in the population aged 80 and older, a decrease in average comorbidities is observed, which contradicts the prevailing literature on multimorbidity phenomena (37, 39, 38).

Among the most prevalent and clinically relevant conditions in various age groups, we consistently find Chronic kidney disease, unspecified (N18.9), Other specified congenital malformations of the heart (Q24.8), Other specified forms of chronic ischemic heart disease (I25.8), Heart failure, unspecified (I50.9), Unspecified atherosclerosis (I70.9), and Other specified cardiac arrhythmias (I49.8). This prevalence may stem from the interconnected nature of these conditions within the network, where several relationships involve equally significant diseases, influencing various physiological processes (1). However, it is crucial to note that the relevance of some of these conditions within the network may diminish or be absent in certain age groups, contingent on the SES of the patients, as we will discuss later.

This observation that younger patients of low socioeconomic status are more likely to have comorbidities than older subjects of higher SES raises relevant questions. Some of these issues may be related to limited access to healthcare, social determinants of health in early life, nutrition and lifestile factors, environmental factors, educational attainment and healthcare utilization patterns, among other constraints (41, 40). Since individuals with lower SES often face barriers in accessing healthcare services, including preventive care and early diagnosis, this may result in undiagnosed or untreated health conditions, contributing to the development of comorbidities. Also, early childhood experiences and SDHs, such as nutrition, access to quality education, and living conditions, significantly influence health outcomes later in life (42, 43). Younger patients from low SES backgrounds may have experienced adverse childhood conditions that contribute to the development of health issues and comorbidities. Younger individuals with lower SES may have limited access to healthy food options, leading to dietary habits that increase the risk of conditions such as obesity, diabetes, and cardiovascular diseases (44, 45). Living in socioeconomically disadvantaged neighborhoods can expose individuals to environmental factors that contribute to poor health outcomes. Environmental stressors, pollution, and lack of recreational spaces may impact the overall health of younger individuals from low SES backgrounds (46).

In summary, a complex interplay of socioeconomic, environmental, and lifestyle factors contributes to the observation that younger patients of low SES are more likely to have comorbidities. These factors highlight the importance of addressing social determinants of health and implementing interventions that promote health equity and access to comprehensive healthcare services for all individuals, regardless of socioeconomic status. Let us examine these complex comorbidity patterns in more detail.

### 4.2 Comorbidity networks in individuals aged 0–20 years

In this initial age range, our analysis of the PRS initially highlighted two diseases that could be associated with low SES. First, *Unspecified heart failure* (I50.9), listed only among the top five in high SES according to Table 2, was revealed through a deeper analysis to maintain a prominent position in low SES, ranking among the top ten most important. This suggests that it is not exclusive to the high SES population (47).

In contrast to *Unspecified heart failure* (I50.9), *Chronic kidney disease unspecified* (N18.9) predominantly affects men with low SES according to our results. There is evidence linking low SES to a predisposition to chronic diseases, including *Chronic kidney disease unspecified* (N18.9), either directly or as a consequence of preceding chronic diseases, with social determinants of health playing a crucial role (48, 49). While further investigation is necessary, factors such as education may be related, suggesting that in this age range, the influence of this factor could stem from the family nucleus where infants and adolescents develop (51, 50). Additionally, habits related to nutrition and lack of physical activity can impact the development of conditions closely related to N18.9, such as obesity (52, 53), a significant health issue in Mexico from an early age (54, 55).

It is worth noting that the presence of Chronic Kidney Disease unspecified (N18.9) in the early years of life is related to congenital malformations and glomerulopathies as the main known causes (57, 56). These conditions may be linked to factors inherent to urbanization, overpopulation, and hygiene, which can negatively impact certain biological processes and increase the risk of developing these diseases (58).

After the above and understanding that being *Chronic Kidney Disease unspecified* (N18.9) a condition that mostly affects low-SES men in this age range, its comorbidities will also may be specific to this population. Therefore, through an analysis of its first neighbors, we decided to examine its relationship with *Systemic Lupus Erythematosus with organ or system involvement* (M32.1), since they have a somehow direct relationship (59) and, in turn, it is also a disease that we can more commonly find in low-SES men (60, 61) according to our findings.

Given that *Chronic Kidney Disease unspecified* (N18.9) predominantly affects low-SES men in this age range, its comorbidities are likely specific to this population. Therefore, through an analysis of its first neighbors, we decided to explore its relationship with *Systemic Lupus Erythematosus with organ or system involvement* (M32.1), as they have a somewhat direct relationship (59). Moreover, it is also a disease more commonly found in low-SES men (60, 61) according to our findings.

Regarding this pair of diseases, lupus erythematosus tends to affect various vital organs, and although less frequent in children, it is more severe than in adults, with kidney disease present in 50%–90% of patients. Therefore, the close relationship between these conditions within the network is not surprising. The association of M32.1 with low SES may be attributed to the condition’s multifactorial nature, involving genetic and environmental factors. Recurrent infections are also known risk factors for triggering the onset of the disease, with these types of infections more prevalent in families where young children live with school-aged children. Such situations are characteristic of overcrowded environments where multiple families coexist, a scenario more common among individuals with low SES. Premature or low birth weight babies, found more frequently in low-SES settings, are also significant factors in recurrent infections (62).

Conversely, in women, *Chronic Kidney Disease unspecified* (N18.9) does not exhibit different impacts by SES, according to our data. This suggests that differences in its occurrence by SES may be less pronounced in this population, although further research is needed to confirm this. Additionally, the relationship with Systemic Lupus Erythematosus with organ or system involvement (M32.1) is present irrespective of SES. This may be related to the fact that women are more predisposed to developing M32.1. However, it’s essential to consider that both diseases are linked to cardiovascular system issues, influenced by biological and lifestyle factors, aligning with the context of the data from which these networks were modeled. Therefore, other associated variables need to be considered to ascertain the significance of SES in the concurrent occurrence of these conditions in men.

### 4.3 Comorbidity networks in individuals aged 21–40 years

In the networks specific to individuals aged 21–40 years, we observe that although the same diseases maintain their top positions according to their Importance Page Rank, the relationships some diseases have with their first neighbors vary. An illustrative example is the case of *Other specified congenital malformations of the heart* (Q24.8), which, exclusively in low SES for both men and women, retains a first-neighbor relationship with *Chronic kidney disease, unspecified* (N18.9) and *Other forms of chronic ischemic heart disease* (I25.8), a relationship suggested in previous literature (64, 63).

The multifactorial etiology of Q24.8 as a congenital malformation implies potential influences from genetic and maternal factors during pregnancy, including maternal health conditions such as diabetes, hypertension, and obesity (67, 65, 66, 68, 69). On the other hand, N18.9 and I25.8 share common factors associated with chronic diseases, such as unhealthy diets and sedentary lifestyles, believed to be more prevalent in low SES populations (70–72).

Thus, the shared social determinants of these three diseases in low SES, including the presence of unhealthy habits, a family history of chronic diseases, and limited access to healthcare, could contribute to their co-occurrence in this population. While further research is necessary to confirm this relationship, the current study highlights the significant association between the concurrent occurrence of these diseases and the mutual information they share in their respective networks.

A more specific and noteworthy case pertains to the association between *Chronic Kidney Disease, unspecified* (N18.9) and *Other specified chronic ischemic heart disease* (I25.8), observed exclusively in the men network of low SES individuals within this age range. This relationship is characterized by a significant MI score of 0.021196, indicative of its clinical relevance as a comorbidity in this population (74, 73). The co-occurrence of these two diseases is anticipated due to the well-established predisposition of kidney disease to cardiovascular conditions, with I25.8 being a notable example. Furthermore, the incidence of I25.8 is known to be age- and sex-related, with a lower likelihood of development among women of childbearing age due to the protective effect of sex hormones (75). In this context, an intermediate disease, *Other specified congenital malformations of the heart* (Q24.8), could partially explain the observed association between N18.9 and I25.8. However, additional research is necessary to confirm this hypothesis. Notably, the exclusive appearance of this comorbidity in the low SES men network is likely linked to shared structural and lifestyle factors discussed earlier.

### 4.4 Comorbidity networks in individuals aged 41–60 years

The most relevant conditions within the networks for high SES remain consistent in the top five positions for both men and women. There are striking similarities in the lower SES, where *Chronic kidney disease, unspecified* (N18.9) replaces *Heart failure, unspecified* (I50.9). Nevertheless, the latter remains among the top ten most important conditions (i.e., high Page Rank Score) according to our results. Hence, among the most significant differences observed in this age range, it is notable that men, irrespective of SES, exhibit a first-neighbor relationship among all diseases included in the top five networks for men in this age range (see Table 4). In contrast, women, as per our data, manifest different configurations contingent on their SES. Notably, only a direct relationship between *Atherosclerosis, unspecified* (I70.9) and *Chronic kidney disease, unspecified* (N18.9) is evident in women of high SES. This pairing becomes noteworthy because it is the sole age range where a discrepancy surfaces concerning sex and SES. It suggests that solely women of high SES exhibit this comorbidity at earlier ages than those of low SES (76), unlike men who experience this comorbidity in both SES.

Regarding this pair of diseases, it is established that *Chronic kidney disease* (N18.9) tends to foster the development of cardiovascular diseases, including *Atherosclerosis* (I70.9), due to deficiencies intrinsic to renal deterioration and its association with the cardiovascular system. This association becomes more prominent in advanced stages of renal disease (78, 77), underscoring the close relationship between both conditions. The differentiated appearance in women based on SES, affecting initially women of high SES, is a counterintuitive phenomenon. Typically, chronic diseases are anticipated to emerge earlier or with more substantial impact in low SES populations due to the interplay of various factors inherent in low SES (81, 82, 80, 79). Further research is warranted to comprehensively understand this phenomenon.

### 4.5 Comorbidity networks in individuals aged 61–80 years old

Regarding this age range, in general, the diseases that occupy the top positions in each network remain constant, according to their PRS, presenting some changes in terms of their level of relevance according to Table 5. That being said, we analyzed how the most relevant diseases were organized with others, present in the aforementioned table with the highest PRS, which showed us that *Rheumatic diseases of endocardial valve unspecified* (I09.1) only appears significantly connected to *Other specified congenital malformations of heart* (Q24.8) in low SES men (83, 84). While this association may be expected given that cardiac malformations could contribute to the development of I09.1, the exclusive appearance of this relationship in men of low SES is noteworthy.

In addition to the presence of a cardiac malformation, other risk factors that are associated with I09.1, such as poor oral health and hygiene (85, 86) or injectable drug use (87), may increase the likelihood of co-occurrence between these diseases in individuals of low SES. Studies have linked poor dental hygiene to low SES, which may result from limited access to education or health services (88, 90, 89). Likewise, low SES has been associated with higher drug consumption, possibly due to factors such as education, family background, place of residence, and social relationships (91). Injectable drug use has been specifically linked to poverty and unemployment, although this relationship requires further investigation (92).

As can be seen in the previous paragraph, the joint presence of the aforementioned conditions, despite being influenced by biological factors, the weight in the occurrence of *Unspecified rheumatic diseases of endocardial valve (I09.1)* may be falling on the conditions that people go through throughout their development, giving rise to generating comprehensive interventions in the treatment and care of patients with congenital malformations. These interventions should not only focus on medical treatment, but also on the care of vulnerable groups, in order to reduce the gaps in inequality that could be influencing why both conditions affect more people with low SES (84, 93).

### 4.6 Comorbidity networks in individuals aged 80 years and older

In this population, we found that despite the fact that the most relevant diseases in the different networks remain constant, the relationships they form between them can be different. An example of this is the one presented by *Acute transmural anterior wall myocardial infarction* (I21.0) and *Unspecified rheumatic valve diseases* (I09.1), as they only showed a relationship in low-SES women in this age range (95, 94, 93).

Regarding this pair, the literature generally indicates that *Acute transmural anterior wall myocardial infarction* (I21.0) is a rare or infrequent complication in patients with *Unspecified rheumatic valve diseases* (I09.1), occurring in the acute phase of the disease, where coronary embolism is related to bacterial endocarditis, thus causing an acute myocardial infarction (96). This helps us to confirm that there may be biological processes involved in the co-occurrence of these two conditions, but leaves their relationship with low SES up in the air, so it is necessary to continue investigating this topic and also to analyze why it appears more frequently in women since, as mentioned above (97), both diseases have a strong and well-positioned relationship according to our results. This could become more important if we take into account that mortality from *Acute transmural anterior wall myocardial infarction* (I21.0) increases directly with people’s age (98), and according to our results, both are very well-positioned diseases in the network of women over 80 years old according to their PRS (Table 6).

### 4.7 Cardiovascular comorbidity in the context of social determinants of health

Social determinants of health are the conditions in which people are born, grow, live, work, and age, and they play a crucial role in shaping health outcomes. These determinants are influenced by the distribution of money, power, and resources at global, national, and local levels. The main SDHs include socioeconomic stutus, education, employment and working conditions, social support networks, healthcare access and quality, physical environment, social and economic policies, cultural and social norms, early childhood experiences and behavioral factors. Understanding and addressing these social determinants are essential for developing effective public health policies and interventions aimed at improving overall health and reducing health disparities (99).

In the present context, our findings just presented point out to some general trends. More specific issues may be found by using the comorbidity network to navigate on local hospital EHRs that include personal patient data. Since that data is to be protected by confidentiality and ethical constraints here we will focus on the general associations. For instance, notable associations with age include congenital heart malformations in younger age groups and an increased prevalence of conditions like chronic kidney disease and atherosclerosis in older age groups. Distinct patterns are observed in men and women, such as the relationship between chronic kidney disease and atherosclerosis being significant in women but more nuanced in men. Differences in disease associations are identified based on SES, highlighting conditions more prevalent in low SES populations, such as chronic kidney disease and rheumatic diseases. In terms of multifactor risks, study delves into the intersectionality of age, sex, and SES, examining how these factors collectively shape cardiovascular comorbidity patterns.

Our findings reveal nuanced relationships, such as the appearance of specific comorbidities in women of high SES at earlier ages, emphasizing the need for a comprehensive understanding of social determinants’ interplay. The identified comorbidity patterns offer insights into potential interventions that consider the social determinants of health. Addressing structural factors like oral health, hygiene, and drug use may be crucial for managing specific comorbidities associated with low SES. Comprehensive interventions focusing on vulnerable groups, especially those with congenital malformations or several risk-related SDHs, are proposed to reduce health inequalities. The study underscores the necessity for continued research to deepen the understanding of the observed relationships, especially those involving age, sex, and SES.

It is relevant however to point out that in this work, statistically significant associations are presented, but no causal or mechanistic explanations have been developed. Rather our study aims to be a starting point to study these, as well as a tool to inform hospital management and public health officials to help them in planning and policy development if possible.

### 4.8 Summary of findings

In what follows we will summarize the more relevant, general results observed by examining the comorbidity and multimorbidity patterns. These global (in the context of our analyzed populations) trends may help contextualize the highly variable landscape of cardiovascular comorbidity presented in this study and available at the Supplementary materials (i.e., the whole set of CVC networks).

• **Comorbidity networks in people aged 0–20 years old**
  • Unspecified Heart Failure (I50.9)
  • Initially associated with high SES but remains important in low SES.
  • Indicates it is not exclusive to high SES populations.
  • Chronic Kidney Disease Unspecified (N18.9)
  • Primarily affects low-SES men.
  • Linked to low SES through factors like education, nutrition, and lifestyle.
  • Relationship with Systemic Lupus Erythematosus (M32.1) explored.
  • Chronic Kidney Disease (N18.9) and Systemic Lupus Erythematosus (M32.1)
  • Lupus affects vital organs, often co-occurs with kidney disease.
  • Shared risk factors include infections, crowded living conditions, and low SES.
• **Comorbidity networks in people aged 21–40 years old**
  • Other Specified Congenital Malformations of the Heart (Q24.8)
  • Connected to Chronic Kidney Disease Unspecified (N18.9) and Other Forms of Chronic Ischemic Heart Disease (I25.8).
  • Shared social determinants, including unhealthy habits and limited healthcare access.
  • Chronic Kidney Disease (N18.9) and Other Forms of Chronic Ischemic Heart Disease (I25.8)
  • Specific to low-SES men.
  • Reflects the known association between kidney disease and cardiovascular conditions.
• **Comorbidity networks in people aged 41–60 years old**
  • Chronic Kidney Disease Unspecified (N18.9)
  • Replaces Unspecified Heart Failure (I50.9) in high SES.
  • Key comorbidity with Atherosclerosis (I70.9) in high-SES women.
  • Suggests SES-related differences in disease patterns.
• **Comorbidity networks in people aged 61–80 years old**
  • Rheumatic Diseases of Endocardial Valve Unspecified (I09.1)
  • Connected to Other Specified Congenital Malformations of the Heart (Q24.8) in low-SES men.
  • Factors include cardiac malformations, poor oral health, and injectable drug use.
• **Comorbidity networks in people aged 80 years and older**
  • Acute Transmural Anterior Wall Myocardial Infarction (I21.0) and Unspecified Rheumatic Valve Diseases (I09.1)
  • Relationship observed in low-SES women.
  • Rare complication, biological processes involved.
  • Need for further investigation into SES-related and gender-related aspects.

### 4.9 Relation to other studies

Mapping the comorbidity and multimorbidity landscape of cardiovascular diseases has been an issue of interest in the international medical and biomedical research community for some time. Different approaches parallel and complementary to what we have just presented have been developed. These efforts span from the highly specific to the very broad approaches. One quite relevant example of the latter is *MorbiNet*, a Spanish study that analyzes a very large population consisting of 3,135,948 adult people in Catalonia, Spain. This work also mined EHRs but is focused exclusively in the relationship between common chronic conditions and type 2 diabetes (100). MorbiNet, as the present work is a network-based approach; there the authors build networks from odds-ratio estimates adjusted by age and sex and considered connections those conditions with odds ratios larger than 1.2 and Bonferroni-adjusted logistic regression p-values less than 1E-5. This work reports that the more connected conditions in T2DM undirected network were: complicated hypertension and atherosclerosis/peripheral vascular disease, cholecystitis/cholelithiasis, retinopathy and peripheral neuritis/neuropathy. The strongest associations from T2DM directed networks were to retinopathy (OR: 23.8), glomerulonephritis/nephrosis (OR: 3.4), peripheral neuritis/neuropathy (OR: 2.7) and pancreas cancer (OR: 2.4). Though their methods are in some sense similar to ours, there are some noticeable differences. Perhaps the most evident is that due to the large scale of their study they focus on common chronic diseases, somehow regardless of the outcomes and mainly related to one (admittedly extremely important) condition, type 2 diabetes. Also, their networks are unweighted, meaning that every comorbidity relationship above the significance threshold contributes to the comorbidity landcape on a similar fashion, whereas in our case, every comorbidity relationship is characterized by a mutual information value representing the relative strength of this association.

Though not exactly a comorbidity analysis, the framework to study cardiovascular diseases from the standpoint of network science as presented by Lee and coworkers is worth mentioning (101). There, the authors establish a set of basic network theory principles that allowed them to look up to disease-disease interactions, uncovering disease mechanisms, and even allow for clinical risk stratification and biomarker discovery. A simialr approach is sketched by Benincassa and collaborators (102) though the scope is more limited to uncover disease modules.

A hybrid network analytics/classical epidemiology approach is presented by Haug et al. (103). They analyzed multimorbidity patterns, representing groups of included or excluded diseases, delineated the health states of patients in a population-wide analysis spanning 17 years and encompassing 9,000,000 patient histories of hospital diagnoses (a data set provided by the Austrian Federal Ministry for Health, covering all approx. 45,000,000 hospital stays of about 9,000,000 individuals in Austria during the 17 years from 1997 to 2014.). These patterns encapsulate the evolving health trajectories of patients, wherein new diagnoses acquired over time alter their health states. Their study assesses age- and sex-specific risks for patients to acquire specific sets of diseases in the future based on their current health state. The population studied is characterized by 132 distinct multimorbidity patterns. Among elderly patients, three groups of multimorbidity patterns are identified, each associated with low (yearly in-hospital mortality of 0.2%–0.3%), medium (0.3%–1%), and high in-hospital mortality (2%–11%). Combinations of diseases that significantly elevate the risk of transitioning into high-mortality health states in later life are identified. For instance, in men (women) aged 50–59 with diagnoses of diabetes and hypertension, the risk of entering the high-mortality region within one year is elevated by a factor of 1.96 ± 0.11 (2.60 x00B1; 0.18) compared to all patients of the same age and sex, respectively. This risk increases further to a factor of 2.09 ± 0.12 (3.04 ± 0.18) if they are additionally diagnosed with metabolic disorders. This study is simialr to ours in the sense that was not limited for particular diagnosis (though only considered 1,074 codes from A00 to N99, grouped into 131 blocks as defined by the WHO, which excludes congenital diseases that are quite relevant for children and young individuals) and it was based on mining ICD-10 codes from the EHRs. Their emphasis however, is different from ours since thay are more interested in *patient trajectories* which describe the health state of this patient at different points in time, rather than in general trends useful for hospital management.

### 4.10 Limitations of the present study

This study utilizes ICD-10 codes to document and classify disease conditions. It is important to note that the use of ICD-10 codes in research presents challenges and limitations, as the system was primarily developed for hospital administration and cost-estimation purposes, rather than as a controlled vocabulary for standardized clinical reporting or epidemiological research (1). Concerns about the suitability of ICD codes for other secondary purposes, such as research or policy interventions, have been raised due to coding errors found in patient data by some authors (105, 106, 104, 107).

The validity of ICD codes to identify specific conditions depends on the extent to which the condition contributes to health service use, as well as the time, place, and method of data collection (108). Diagnostic accuracy tests of ICD-10 codes have been conducted to evaluate features such as sensitivity, specificity, positive predictive values (PPV), and negative predictive values (NPV) for specific major diagnoses, major procedures, minor procedures, ambulatory diagnoses, co-existing conditions, and death status. These studies have generally found good-to-excellent coding quality for ICD-10 codes in these areas (1).

Given these considerations, when using these codes for clinical purposes, careful evaluation is necessary since the actual subjects of interest may not be accurately defined. This may be critical in the assessment of chronic conditions. Moreover, ICD codes perform better with sets of diseases enriched for frequent, well-known conditions.

It is noteworthy that in the specific case of Electronic Health Records in the NICICH, the administrative database coding, archiving, and retrieval procedures have been certified and validated by the World Health Organization (WHO) through the local ‘Collaborating Center for WHO International Classification Schemes - Mexico Chapter’ (CEMECE, for its Spanish acronym). These procedures are in agreement with ISO 9001:2000, ISO/IEC 27001 certifications, and with the Official Mexican Norm (NOM for its Spanish acronym): NOM-004-SSA3-2012 (1).

## 5 Concluding remarks

In conclusion, the analysis of comorbidity networks across different age groups and socioeconomic status reveals interesting patterns in disease co-occurrence. There are consistent associations between certain diseases, and these associations may vary based on age and SES. Moreover, the presence of certain comorbidities differ between men and women and across different age and SES, as expected.

Some diseases, such as chronic kidney disease and specific cardiac conditions, consistently appear among the most relevant comorbidities across age groups and SES. Additionally, the study highlights specific associations, such as the relationship between unspecified heart failure, chronic kidney disease, and systemic lupus erythematosus with organ involvement, which may have implications for diagnostic and therapeutic strategies.

Notably, the findings also suggest that individuals with low SES tend to exhibit a greater diversity of diseases, potentially indicating disparities in health outcomes and access to healthcare resources. The importance of social determinants of health in shaping comorbidity patterns is evident, emphasizing the need for comprehensive interventions that address not only medical aspects but also social and environmental factors.

Overall, this study provides valuable insights into the complex landscape of comorbidities, shedding light on how age, sex, and SES contribute to the interconnected web of diseases. Further research and ongoing investigation are crucial to deepen our understanding of these relationships and inform more targeted and effective approaches to healthcare and disease prevention.

## Data Availability

The data analyzed in this study is subject to the following licenses/restrictions: Data was taken from annonymized Electronic Health Records from the National Institute of Cardiology Ignacio Chavez. Data summaries are available upon request. Requests to access these datasets should be directed to mireya.martinez@cardiologia.org.mx.
